# Gender differences in income among psychiatrists in China: Findings from a national survey

**DOI:** 10.3389/fpubh.2022.1026532

**Published:** 2022-12-05

**Authors:** Xinxin Han, Lijun Shen, Jiayu Tong, Feng Jiang, Huanzhong Liu, Jiming Zhu

**Affiliations:** ^1^School of Public Health and Emergency Management, Southern University of Science and Technology, Shenzhen, China; ^2^Vanke School of Public Health, Tsinghua University, Beijing, China; ^3^School of Medicine, Tsinghua University, Beijing, China; ^4^Institute for Hospital Management, Tsinghua University, Shenzhen, China; ^5^School of International and Public Affairs, Shanghai Jiao Tong University, Shanghai, China; ^6^Institute of Healthy Yangtze River Delta, Shanghai Jiao Tong University, Shanghai, China; ^7^Department of Psychiatry, Chaohu Hospital of Anhui Medical University, Hefei, China; ^8^Department of Psychiatry, Anhui Psychiatric Center, Anhui Medical University, Hefei, China; ^9^Institute for Healthy China, Tsinghua University, Beijing, China

**Keywords:** income difference, gender difference, psychiatrists, health workforce, China

## Abstract

**Background:**

Gender income disparity in healthcare settings is a longstanding issue around the globe, but such evidence among Chinese psychiatrists is scarce. This study investigated whether gender income differences exist among physicians in China.

**Methods:**

Data came from the 2019 national survey data of 4,520 psychiatrists in major public psychiatric hospitals across China. Self-reported monthly income after tax (in Chinese Yuan, CNY) by participants at all professional ranks was assessed. Average monthly income by gender was reported. Adjusted income differences between male and female psychiatrists were examined using multivariable regression models, adjusting with inverse probability of treatment weights and controlling for psychiatrist demographics (e.g., gender, professional rank, marital status, educational level, and work hours) and hospital fixed effects.

**Results:**

The unadjusted mean difference in monthly income after tax by gender was 555 CNY (about $86; 95% CI, −825 to −284; mean [SD] for men: 8,652 [4,783] CNY and for women: 8,097 [4,350] CNY) in all psychiatrists. After regression adjustments, the income difference by gender among all psychiatrists reduced substantially and became insignificant. However, gender income difference was still observed among senior-level psychiatrists, where female psychiatrists earned 453 CNY (about $70; 95% CI, −810 to −95) significantly less than male psychiatrists.

**Conclusion:**

China achieved gender equity in income for psychiatrists overall, the observed income differences among senior level psychiatrists, however, reveal the persistence of gender inequity at the highest level of professional hierarchy. These findings call for policy attention to the issue of gender income disparity among psychiatrists in China's healthcare system.

## Introduction

Gender income disparity in healthcare settings is a longstanding issue around the globe. In many countries, female physicians have often been demonstrated to earn significantly less than their male peers (1–5). However, publicly available data on physician income in China is scarce, not even mention evidence about gender income disparity. To the best of our knowledge, there was only one national survey that systematically investigated physician salaries in China (6). The study found a salary difference between female and male physicians, although such a difference became insignificant after regression adjustments. Another regional survey study of physicians in county-level healthcare facilities in rural western China also observed descriptively income differences between female and male physicians (7).

There are many factors contributing to income differences, such as age, marital status, educational level, specialty, work hours, and administrative position (3, 5, 8–10). In China's healthcare system, professional rank, a recognition of the level of technical expertise and work ability for healthcare professionals, is also associated with physician income (6). Similar to the faculty rank that is more commonly used in the globe, professional rank for physicians in China contains three levels: junior-level (i.e., often new graduates and residents), middle-level (i.e., attending physicians), and senior-level (i.e., chief physicians who are exceptional experts of their specialty and have reached the highest level of professional hierarchy in their areas). Senior-level physicians usually have longer work years and are often in leadership positions (some may also be in a management position, such as department chair or vice chair, which may allow them to earn more than their peers).

Like other leadership positions or occupational ranks, gender inequity exists in this professional hierarchy, with women less likely to be promoted to the senior level (6). Even among physicians at the same rank, such kind of gender inequity is likely to contribute to gender income differences. In the United States, gender differences in salary were found at all faculty ranks and were the largest among full professors among academic physicians (11). Another study also revealed gender differences in salaries of clinical department chairs in US public medical schools, with women earning less than men (12). Whether gender income differences exist at the highest level of professional hierarchy in China's healthcare system is, however, unknown.

In this study, we focused on exploring income differences among psychiatrists—the specialty that has not yet been examined in any China's physician income studies. Even globally, evidence on gender income differences among psychiatrists is scarce, and the data used in existing studies were mostly out-of-date (13); for example, a study published in 2007 used 1992–2001 data to examine gender differences in annual income of psychiatrists in the United States (14). Therefore, in this study, we used more recent data from a national survey of psychiatrists in 41 psychiatric hospitals across China to explore whether gender income differences exist among Chinese psychiatrists, and in particular to investigate whether such differences exist among psychiatrists at the senior level. Findings of this study can add to existing evidence on income difference of psychiatrists in China, as such evidence is not currently available in healthcare research, and calls for policy attention to the issue of income disparity in China's context.

## Methods

### Data and study participants

We retrieved data from the supplement of the National Hospital Performance Evaluation Survey sponsored by China National Health Commission in 2019. The supplement was conducted from March 18 to 31 in 2019, and collected individual information of 4,708 psychiatrists from 41 psychiatric hospitals across 29 provinces in China, including participants' demographic characteristics, work status, and other workforce-related information. The questionnaire was distributed and filled anonymously *via* a widely used smartphone application in China. Informed consent was taken from every participant before the survey and personal privacy was completed protected during the whole study process.

A total of 4,520 psychiatrists completed the survey, yielding a response rate of 96%. In China, almost 90% psychiatrists work in psychiatric hospitals. Because the 41 psychiatric hospitals covered all tertiary psychiatric hospitals in China, our study sample should be taken into account as nationally representative of China's psychiatrists from tertiary hospitals.

### Outcome

The outcome of interest was the self-reported monthly income after tax (in Chinese Yuan, CNY) by participants. The survey asked participants to report their income after tax, which included the basic salary, benefits, bonuses, medical practice earnings, and other compensations.

### Control variables

The following available participants' demographic information were included in the analysis as control variables in the regression analysis: gender (female vs. male), professional rank (junior, middle, and senior), marital status (married vs. unmarried), educational level (bachelor's degree or below vs. master's or doctoral degree), work hours per week, years of practice, whether being at an administrative position (e.g., department chair or vice chair), and whether working in outpatient settings. These variables were included based on data availability and prior literature that indicated their associations with physician income (3, 5, 8–10, 14).

### Statistical analysis

Since the income data were self-reported by participants, outliers may exist due to input error or recall bias. To reduce the impact of outliers on data accuracy, we cleaned the income data by dropping outliers that were outside quartile 1 and 99 (*N* = 70), as did in other studies that used income data (15, 16). Our final analytical sample included 4,450 individual psychiatrists. We reported the average monthly income and income differences for female and male psychiatrists by different professional ranks.

To estimate the adjusted gender income differences, we constructed a multivariable linear regression model, where the self-reported monthly income was the outcome variable, and gender was the key explanatory variable. To account for differences in demographic characteristics between female and male psychiatrists, we used the propensity score weighting approach. The specific method we used was the inverse probability of treatment weighting, where weights were calculated using the estimated propensity scores. The propensity score was the probability of a participant being in the treatment group (in this study, the female group), given its observable baseline characteristics, and used to balance all observable characteristics. The propensity scores were estimated using the following characteristics: professional rank, marital status, educational level, administrative position, whether having outpatients, work hours per day, and years of practice. These variables were available in the dataset and could affect the probability of being a female while contributing to differences in income. We calculated the inverse probability of treatment weights using the propensity scores, in which the weight was equal to the inverse probability of receiving the treatment that was actually received (17, 18). Thus, the weight for females was 1/(propensity score) and the weight for males was 1/(1-propensity score). We stabilized the weights to a mean of one and trimmed at the 99th percentile. After applying the inverse probability weights, we successfully balanced all but one of the observable characteristics within our weight sample (years of practice), which we directly controlled for in our model. This approach creates a synthetic distribution of female and male participants, where female and male participants are weighted to be as similar as possible based on observable demographic characteristics, and thus approximates a randomized experiment by isolating the effects caused by gender difference rather than the effects by underlying differences between groups (19, 20).

We applied the inverse probability of treatment weights to the regression model and also directly controlled for participant characteristics that were used in generating the propensity scores. We also included hospital fixed effects in the model to account for hospital-level variations (such as hospital size, location, workforce, and patient volumes). Standard errors were clustered by participants' working hospital to account for autocorrelation among participants. We repeated the model for junior-, middle-, and senior-level psychiatrists. Significance level was set at 0.05 with two-tail tests. All analyses were performed using Stata version 16 (StataCorps, Inc.).

## Results

### Characteristics of study participants

Of the 4,450 participants, 2,591 (58%) were female (Table 1). A total of 1,370 (31%) were junior psychiatrists, 1,536 (35%) were middle-level psychiatrists, and 1,544 (35%) were senior psychiatrists. More females were at the junior level, unmarried, had a master or doctoral degree, were less likely to be at an administrative position, and have fewer years of practice compared to male participants.

**Table 1:** Participant characteristics.

**Characteristics**	**No. (%)**		***P*-value^a^**
	**Male**	**Female**	
Total no.	1,859 (41.8)	2,591 (58.2)	NA
**Professional rank**			<0.01
Junior level	480 (25.8)	890 (34.3)	
Middle level	629 (33.8)	907 (35.0)	
Senior level	750 (40.3)	794 (30.6)	
**Marital status**			<0.01
Married	1,584 (85.2)	2,036 (78.6)	
Unmarried	275 (14.8)	555 (21.4)	
**Educational level**			<0.01
Associate degree or below	69 (3.7)	122 (2.0)	
Bachelor's degree	1,260 (67.8)	2,814 (60.0)	
Master's or doctoral degree	530 (28.5)	1,514 (38.0)	
**Administrative position**			<0.01
No	1,325 (71.3)	2,187 (84.4)	
Yes	534 (28.7)	404 (15.6)	
**Having outpatients**			<0.01
No	620 (33.4)	1,017 (39.3)	
Yes	1,239 (66.6)	1,574 (60.7)	
**Work hours per day**			
Mean (SD)	9.41 (2.29)	9.31 (1.94)	0.12
**Years of practice**			
Mean (SD)	16.30 (10.05)	12.66 (9.07)	<0.01

NA, not applicable.

a P-values were from Pearson Chi-square tests comparing percentages or from two-tail t-tests comparing means between men and women.

### Unadjusted and adjusted results of gender differences in income

Overall, the average monthly income after tax was 8,097 (SD = 4,350) CNY for Chinese female psychiatrists and 8,652 (SD = 4,783) CNY for male psychiatrists (Table 2). The unadjusted mean difference in income was significant by gender in all psychiatrists (555 CNY, about $86; 95% CI, −825 to −284). However, this difference reduced substantially and became insignificant after adjustments (income difference = −31, 95% CI = −206 to 143).

**Table 2:** Multivariable linear regression analyses on gender differences in income, adjusted with inverse probability of treatment weights.

	**Monthly income, in Chinese** **Yuan** **Mean (SD)**		**Gender differences in income** **[F–M] (95% CI), in Chinese Yuan**			
	**Female**	**Male**	**Unadjusted**	***P*-value^a^**	**Adjusted**	***P*-value^b^**
All psychiatrists^c^	8,097 (4,350)	8,652 (4,783)	−555 (−825 to −284)	<0.001	−31 (−206 to 143)	0.72
Junior-level psychiatrists^d^	6,106 (2,759)	5,972 (2,500)	135 (−162 to 432)	0.37	−47 (−252 to 158)	0.65
Middle-level psychiatrists^d^	7,964 (3,677)	7,776 (3,311)	188 (−171 to 548)	0.30	90 (−227 to 407)	0.57
Senior-level psychiatrists^d^	10,480 (5,261)	11,101 (5,684)	−621 (−1168 to −75)	0.03	−453 (−810 to −95)	0.01

F, female; M, male.

a P-values were from two-tail t-tests comparing means between female and male.

b P-values were from regression adjusted with inverse probability of treatment weights. Full regression results were presented in the Appendix Table A1.

c Adjusted for professional rank, marital status, educational level, work hours per week, years of practice, administrative position, working in outpatient settings and hospital fixed effects. Standard errors were clustered by participants' working hospital.

d Adjusted for marital status, educational level, work hours per week, years of practice, administrative position, working in outpatient settings and hospital fixed effects. Standard errors were clustered by participants' working hospital.

We observed gender income differences among senior-level psychiatrists in both unadjusted analysis and adjusted regression. On average, the monthly income was 10,480 (SD = 5,261) CNY for female senior psychiatrists and 11,101 (SD = 5,684) CNY for male senior psychiatrists. The unadjusted mean income difference was significant between female and male psychiatrists (621 CNY, about $96; 95% CI, −1,168 to −75). This difference persisted in the model after we adjusted with the inverse probability of treatment weights and controlled for demographic characteristics and other factors. After adjustments, the mean difference in income by gender among senior-level psychiatrists was 453 CNY (about $70, 95% CI = −810 to −95). We did not observe significant gender income differences in junior- and middle-level psychiatrists, neither in the unadjusted analysis nor adjusted regression. Full regression results were presented in the Appendix Table A1.

## Discussion

This study, for the first time to our knowledge, used recent data from a nationally representative sample to investigate gender income differences among physicians in China. Overall, we observed that significant income differences exist between female and male psychiatrists in the unadjusted analysis but not in the adjusted regression. When we looked at income differences by professional rank, we found significant gender income differences among senior-level psychiatrists, where female earned 453 Yuan (about $70) monthly less than male psychiatrists after regression adjustments. Our findings suggest that although China achieved gender equity in income for psychiatrists overall, gender inequity still persists at the highest level of professional hierarchy, even under the context that women at this rank have demonstrated the same level of technical expertise and work ability in their areas as men.

Our study was observational and cannot answer why such differences exist. A common explanation might be that such differences are rooted in socio-cultural structures that restrict the opportunities of women in the labor market (4). It is possible that even when female physicians have reached the highest level of professional rank, they may still be less likely to receive recognition for achievements than male physicians which restricts their career opportunities. Other socio-cultural factors could also contribute to such differences, for example, differential household responsibilities and childrearing between women and men could lead women to place less emphasis on their career but more on family compared to their male counterparts in subsequent career development (21–23). Future research is warranted to explore in-depth reasons and other factors (such as years of practice) that drive such differences in China's context.

Notably, our study did not find significant gender income differences among psychiatrists at the junior and middle level. It is likely that both female and male physicians at the junior or middle level have less flexible time to leave clinical work, and therefore, have less opportunities to increase their income through other revenues. It is also worth to note that, the income levels for junior and middle level psychiatrists in general are much lower than senior psychiatrists. This issue is critical in China, as many young physicians are turning over healthcare industries because of low income (24–26). Research has also demonstrated that low income is the major reason for turnover intention among China's psychiatric residents (27). Young physicians often have heavier workloads, but workloads and work hours are not tied to income in China's healthcare system. Heavy workloads plus the income that do not match workloads lead to severe turnover issue in healthcare settings, which partly links to shortages of physicians in China (28–30).

Our study calls for policy attention to gender income disparity among senior-level physicians in China's healthcare system. Efforts to monitor and reduce income gaps, for example, can be through publicly reporting salary information and increasing data availability and transparency. In recent years, the Chinese central government has begun to focus on adjusting physician salaries in public hospitals and improving the benefits of physicians in order to stabilize physician workforce (31). Unified governance of physician payment and a more comprehensive compensation plan for senior-level physicians may also minimize gender income gaps.

## Limitations

This study has several limitations. First, the income data are self-reported by survey participants, and we lack actual data on real salary of hospital employees. Participants could likely underreport their income, or the actual income level of psychiatrists in China may be less than what we found because our study participants were from tertiary hospitals that tend to have a higher income level. In future research, it is worth to revisit our study using administrative data such as payroll data or income tax data. Second, since this study only used data at the psychiatric setting, caution must be paid when generalizing our findings to other clinical areas. Third, due to data availability, we were unable to control for other physician productivity, such as research grants, publications and teaching, which are also important source of physician income.

## Conclusion

In this national survey study of a large sample of China's psychiatrists, we did not observe gender income differences among found existing gender income differences among Chinese physicians. However, we did find differences existing among senior physicians who have reached the highest level of professional hierarchy in their areas. These findings call for actions to address such differences in China's healthcare system. Future research is also needed to understand in-depth mechanisms that drive such differences.

## Data availability statement

The datasets presented in this article are not readily available, because it involves privacy information. Requests to access these datasets should be directed to the corresponding author and the National Hospital Performance Evaluation Survey Group (fengjiang@sjtu.edu.cn).

## Author contributions

JZ: conceptualization, methodology, validation, resources, writing—review and editing, and supervision. XH: methodology, formal analysis, and writing—original draft. LS: writing—review and editing. JT: formal analysis. FJ: resources, investigation, and writing—review and editing. HL: resources and investigation. All authors contributed to the article and approved the submitted version.

## Conflict of interest

The authors declare that the research was conducted in the absence of any commercial or financial relationships that could be construed as a potential conflict of interest.

## Publisher's note

All claims expressed in this article are solely those of the authors and do not necessarily represent those of their affiliated organizations, or those of the publisher, the editors and the reviewers. Any product that may be evaluated in this article, or claim that may be made by its manufacturer, is not guaranteed or endorsed by the publisher.

## Appendix

**Table A1:** Multiple linear regression results of gender income differences by professional rank.

	**Total**		**Professional rank**					
			**Junior**		**Middle**		**Senior**	
	**Coff**.	**95% CI**	**Coff**.	**95% CI**	**Coff**.	**95% CI**	**Coff**.	**95% CI**
**Gender**
Female	−31	(−206 to 143)	−47	(−252 to 158)	90	(−227 to 407)	−453^*^	(−810 to −95)
Male	Ref.		Ref.		Ref.		Ref.	
**Professional rank**
Senior	1,388^***^	(1,003 to 1,772)						
Middle	334^*^	(12 to 655)						
Junior	Ref.							
**Marital status**
Married	307	(−37 to 651)	496^***^	(223 to 769)	−104	(−515 to 307)	−692	(−1,393 to 10)
Unmarried	Ref.		Ref.		Ref.		Ref.	
**Educational level**
Master's or doctoral degree	553^*^	(116 to 990)	688^**^	(231 to 1,145)	355	(−25 to 735)	309	(−162 to 779)
Bachelor's degree or below	Ref.		Ref.		Ref.		Ref.	
**Administrative position**
Yes	2,087^***^	(1,694 to 2,479)	1,294	(−1,259 to 3,847)	1,618^***^	(1,035 to 2,201)	2,235^***^	(1,896 to 2,573)
No	Ref.		Ref.		Ref.		Ref.	
**Working in outpatient settings**
Yes	640^***^	(390 to 890)	353^*^	(12 to 694)	538^***^	(304 to 772)	1,111^***^	(575 to 1,647)
No	Ref.		Ref.				Ref.	
Work hours per day	−44	(−96 to 8)	1	(−65 to 66)	−91	(−189 to 8)	−38	(−142 to 66)
Years of practice	84^***^	(53 to 116)	50^**^	(17 to 84)	33^**^	(13 to 52)	113^***^	(79 to 146)
Hospital fixed-effects	Yes		Yes		Yes		Yes	
Observations	4,394		1,345		1,521		1,529	
R-squared	0.601		0.508		0.607		0.576	

Coefficients and 95% confidence intervals (CI) were presented in this table. Standard errors were clustered by hospital. All models adjusted with inverse probability of treatment weights, generating from the propensity score weighting approach.

*** *p* < 0.001,

** *p* < 0.01,

* *p* < 0.05.
